# Automatically Extracted Machine Learning Features from Preoperative CT to Early Predict Microvascular Invasion in HCC: The Role of the Zone of Transition (ZOT)

**DOI:** 10.3390/cancers14071816

**Published:** 2022-04-03

**Authors:** Matteo Renzulli, Margherita Mottola, Francesca Coppola, Maria Adriana Cocozza, Silvia Malavasi, Arrigo Cattabriga, Giulio Vara, Matteo Ravaioli, Matteo Cescon, Francesco Vasuri, Rita Golfieri, Alessandro Bevilacqua

**Affiliations:** 1 Department of Radiology, IRCCS Azienda Ospedaliero-Universitaria di Bologna, Via Albertoni 15, 40138 Bologna, Italy; matteo.renzulli@unibo.it (M.R.); margherita.mottola@unibo.it (M.M.); francesca.coppola@aosp.bo.it (F.C.); mariaadriana.cocozza@studio.unibo.it (M.A.C.); arrigo.cattabriga@studio.unibo.it (A.C.); giulio.vara@studio.unibo.it (G.V.); rita.golfieri@unibo.it (R.G.); 2 Advanced Research Center on Electronic Systems (ARCES), University of Bologna, 40126 Bologna, Italy; s.malavasi@unibo.it; 3 General Surgery and Transplant Unit, IRCCS, Azienda Ospedaliero-Universitaria di Bologna, Sant’Orsola-Malpighi Hospital, 40138 Bologna, Italy; matteo.ravaioli6@unibo.it (M.R.); matteo.cescon@unibo.it (M.C.); 4 Pathology Unit, IRCCS, Azienda Ospedaliero-Universitaria di Bologna, 40138 Bologna, Italy; francesco.vasuri@aosp.bo.it; 5 Department of Computer Science and Engineering (DISI), University of Bologna, 40126 Bologna, Italy

**Keywords:** machine learning, computed tomography, hepatocellular carcinoma, imaging biomarkers, radiomics

## Abstract

**Simple Summary:**

Microvascular invasion (MVI) is a universally recognised predictor of hepatocellular carcinoma (HCC) recurrence after curative treatments, whose diagnosis, nowadays, is still postponed to surgery, through histopathological specimen investigation. This retrospective study aims to exploit a radiomic approach to pre-procedural diagnosis MVI in early-stage HCC, with a diameter ≤ 3 cm. The main novelty of the study is the use of the zone of transition (ZOT), crossing tumour and peritumour, detected adaptively through a standardized procedure based on the analysis of image gradients. After generating radiomics features from ZOT and tumour core in arterial and venous computed tomography phases, a classifier is trained and validated using a signature of four features only. The achieved radiomic model enables the early diagnosis of small HCC (≤3 cm) showing MVI with high specificity (82%) and sensitivity (79%), perspectively providing an effective tool to detect the best candidates for surgical treatment and liver transplantation (negative predictive value = 87%).

**Abstract:**

Background: Microvascular invasion (MVI) is a consolidated predictor of hepatocellular carcinoma (HCC) recurrence after treatments. No reliable radiological imaging findings are available for preoperatively diagnosing MVI, despite some progresses of radiomic analysis. Furthermore, current MVI radiomic studies have not been designed for small HCC nodules, for which a plethora of treatments exists. This study aimed to identify radiomic MVI predictors in nodules ≤3.0 cm by analysing the zone of transition (ZOT), crossing tumour and peritumour, automatically detected to face the uncertainties of radiologist’s tumour segmentation. Methods: The study considered 117 patients imaged by contrast-enhanced computed tomography; 78 patients were finally enrolled in the radiomic analysis. Radiomic features were extracted from the tumour and the ZOT, detected using an adaptive procedure based on local image contrast variations. After data oversampling, a support vector machine classifier was developed and validated. Classifier performance was assessed using receiver operating characteristic (ROC) curve analysis and related metrics. Results: The original 89 HCC nodules (32 MVI+ and 57 MVI−) became 169 (62 MVI+ and 107 MVI−) after oversampling. Of the four features within the signature, three are ZOT heterogeneity measures regarding both arterial and venous phases. On the test set (19MVI+ and 33MVI−), the classifier predicts MVI+ with area under the curve of 0.86 (95%CI (0.70–0.93), *p*∼10${}^{-5}$), sensitivity = 79% and specificity = 82%. The classifier showed negative and positive predictive values of 87% and 71%, respectively. Conclusions: The classifier showed the highest diagnostic performance in the literature, disclosing the role of ZOT heterogeneity in predicting the MVI+ status.

## 1. Introduction

Hepatocellular carcinoma (HCC), the most common primary hepatic malignant tumour, represents a global health problem, and its incidence is increasing worldwide [1,2]. HCC is the third leading cause of cancer-related deaths worldwide and the second leading cause of cancer-specific mortality in the Asia-Pacific regions, especially in China [1,3].

One of the most significant problems plaguing the potentially curative treatments of patients with HCC, such as ablation, surgical resection, or liver transplantation (LT) [4], is the high rate of recurrence which negatively impacts overall survival [5,6]. The cumulative tumour recurrence rates at three-year after the ablation of HCCs ≤ 3.0 cm in patients in the low-, middle-, and high-risk groups were 68.2%, 100%, and 100%, respectively [7]. Five-year HCC recurrence complicates 35% of cases after LT and 70% of cases after hepatic resection [6,8,9,10,11].

Vascular invasion, both macro- and micro-vascular invasion (MVI), is universally recognised as a predictor of recurrence and poor overall survival after treatments for HCC [6,12,13]. However, the pre-treatment clinical significance of the two forms of vascular infiltration is clearly different. Macrovascular infiltration can be correctly diagnosed by imaging in the pre-treatment phase, thus allowing to correctly classify those patients in the advanced stage and direct them to the best treatment, which is systemic therapy [14]. Conversely, MVI is a histologic finding which can only be postoperatively diagnosed with a surgical specimen [13,15], nullifying the potential usefulness of MVI diagnosis in the preclinical phase for selecting patients to submit to systemic therapy, turning MVI into just a post-procedural prognostic factor. In recent years, much effort has been made to identify reliable imaging findings in order to reach an accurate pre-procedural diagnosis of MVI in HCC patients [11,16,17,18,19]. Unfortunately, to date, these criteria for a preoperative radiologic diagnosis of MVI in HCC have not been widely recognised, and thus have not been reported in the current guidelines for the management of HCC [14]. A possible explanation for failing to evolve towards the pre-treatment radiological diagnosis of MVI is that it is merely based on direct observation by radiologists. In fact, to make a diagnosis, radiologists use contrast differences between adjacent tissues which fade in the peritumour where MVI is supposed to most frequently occur [12]. In addition, several aspects may limit the accuracy of their diagnosis, such as basic knowledge, diagnostic experience, and work status, etc. On the other hand, radiological images contain more information than what is visible to the radiologist’s eye; these can be much more informative regarding the tissue of interest than what a radiologist can see [20]. Some progress in the MVI evaluation of HCC has recently been made using radiomics [21,22,23,24]. Moreover, radiomics has emerged as the application of the consolidation of artificial intelligence and machine learning techniques in the radiological field to overcome human limitations. In practice, radiomics relies on the extraction of quantitative features from medical images with the aim of decoding tissue pathology and measuring tumour heterogeneity related to changes in cellularity, necrosis, angiogenesis, and extracellular matrix deposition in the tumour microenvironment [25]. However, radiomic studies dealing with MVI diagnosis have presented several limitations. One of the most important studies is the lack of investigation related to nodule size. According to the most up-to-date guidelines on the management of HCC, the treatments which ensure longer survival, such as ablation, resection, and LT, are dedicated to patients in the very early and early stages of the Barcelona clinic liver cancer (BCLC) staging system [14]. Except for single lesions for which there are no dimensional criteria, the remaining therapeutic choices for patients in the very early and early stages concern nodules, sized ≤3.0 cm in diameter. Moreover, even concerning single lesions, in the era of the ever-increasing extension of the surveillance program for patients at high risk of developing HCC, it is more likely that small lesions will be identified during surveillance. In addition, when analysing the peritumoural area, MVI radiomic studies consider regions relying on the radiologist’s definition of the tumour profile. Furthermore, these peritumoural regions have size and regular shape a-priori defined scarcely fitting natural phenomena. Therefore, it is mandatory, when including the peritumoural area, to overcome the limits of human segmentation by defining the zone of transition (ZOT) between the tumour and the surrounding non-tumour parenchyma, similar to what was done by Zhang et al. [26], thus relaxing the constraints of the defined human profile.

The aim of the present study was to investigate whether radiomic features extracted from contrast-enhanced computed tomography (CT) could predict MVI in a selected population of patients with HCC nodules ≤ 3.0 cm in diameter. The authors also extended the peritumoural analysis to the ZOT, employing a method tailored on purpose, yielding a region with adaptive shape and size.

## 2. Materials and Methods

### 2.1. Study Population

This single-centre retrospective study, involving standard of car carried out at the authors’ tertiary liver care centre, was approved by the Institutional Review Board (N° 197/2020/Oss/AOUBo), and the requirement for informed consent was waived. All procedures involving human participants were performed in accordance with the 1975 Helsinki declaration and its later amendments. The database of the Surgical Unit of the Department of Medical and Surgical Sciences–DIMEC of the IRCCS Polyclinic Sant’Orsola, University of Bologna, Bologna, Italy, was reviewed from January 2014 to December 2018 to identify all patients who underwent hepatic resection for HCC. The patients who satisfied the following criteria were included in the present study: (a) preoperative CT performed in the Authors’ Radiology Unit within three months before surgical resection, (b) an HCC imaging diagnosis reached according to the European Association of the Study of the Liver (EASL) guidelines [14], (c) nodule dimension ≤ 3 cm, and (d) hepatic resection indicated according to the criteria described in a previously published article [27]. Patients who underwent local-regional treatments prior to CT or during the period between CT and hepatic resection were excluded. In the study period, 117 patients with HCC underwent surgery. Of these, 12 who received previous treatments, 24 who had preoperative imaging performed outside the Authors’ radiology unit, and 3 who had inadequate imaging studies were excluded, thus allowing analysis in 78 patients. The protocol requirements for contrast–enhanced CT met the criteria recommended by the EASL guidelines [14]. In detail, the technical specifications of CT were explained in a previously published paper [28].

### 2.2. Histological Data

At the authors’ hospital, all pathologic examinations were performed by a team of pathologists, each one with more than 15 years of experience in liver pathology. Microvascular invasion was defined as a tumour within a vascular space lined by the endothelium, which was visible only at microscopy [12]. The MVI of tumour cells into the portal or hepatic venules and capillaries was pathologically examined by sampling the HCC tissue. For all the HCCs, thanks to having dimensions of ≤3.0 cm, the entire tumour was examined.

### 2.3. Tumour Region Segmentation

Three-dimensional manual segmentation was performed by two experienced radiologists in consensus, using ImageJ software (R1.53d https://imagej.nih.gov/ij/ (accessed on 10 January 2021), a free, public domain software developed by the National Institutes of Health (NIH). Regions of interest (ROIs) were drawn on the arterial-phase and the delayed-phase images, slice-by-slice for each patient, strictly along the visible borders of the lesion to include approximately its entire volume. For the radiomic analysis, all the slices were considered.

### 2.4. Zone of Transition Detection

Recent studies have reported diverse imaging features which have been shown to be reliable predictors of MVI in HCC [18]. Regarding the most relevant radiological signs of MVI, there are the shape, morphology, and contrast-enhancement properties observed at the tumour margins and immediately adjacent outer regions, referred to as the peritumour [21]. For this reason, several radiomic studies for diagnosing MVI have thoroughly considered the peritumoural areas, normally explored by dilating tumour ROIs using standard morphological operators [29]. In effect, there is not clear identification of the ZOT, also due to the uncertainty of the detection of tissues belonging only to the tumour. For this purpose, the definition of ZOT provided in [26] was used to identify the region between the tumour and the surrounding non-tumour tissue. Practically speaking, for ZOT analysis, an ROI crossing tumour borders and including the inner and outer regions was defined. To this end, a two-stage procedure was set up to carry out adaptive ZOT detection, which exploited the image gradient variations analysed at tumour borders.

**Figure 1:**
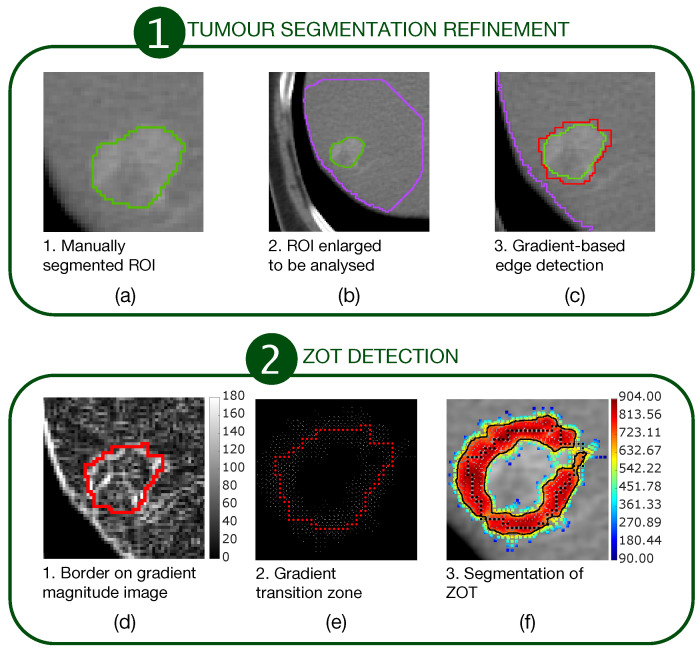
A two-stage procedure for ZOT detection. The first stage (**a**–**c**) refines the manually segmented ROIs. The second stage (**d**–**f**) detects ZOT, which is reconstructed starting from the tumour border, by exploring gradient variations for each pixel of the tumour border.

The first stage consisted of a segmentation refinement process of manually segmented tumour ROIs (Figure 1a–c).

The second stage consisted of ZOT detection based on gradient magnitude analysis, stemming from the tumour border, output from the first stage (Figure 1d–f). Figure 1a outlines a sample tumour ROI (green line), manually segmented. It was then coarsely dilated as shown by the big purple ROI in Figure 1b in order to include a portion of normal liver around the HCC nodule. Edge detection was carried out, based on gradient image variations, searching the directions of the maximum local image gradient. Thus, the red line in Figure 1c shows the refined tumour ROI.

**Figure 2:**
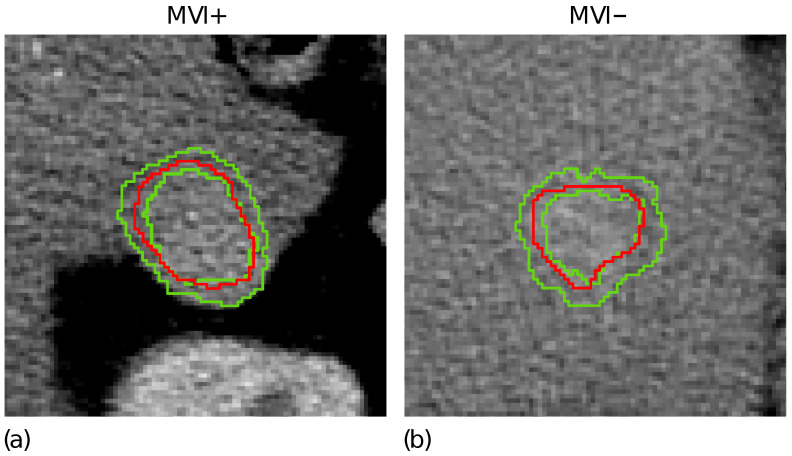
The ZOT is defined between the two green lines for two representative MVI+ (**a**) and MVI− (**b**) nodules; the tumour ROIs are superimposed in red.

Figure 1d highlights the tumour border on the gradient magnitude image. The gradient variations along the gradient direction as well as at ±45 were then analysed. Gradient lines are expected to be bell-shaped curves with a maximum in correspondence of the edge pixel and two local minima on the left and right tails, respectively. Thus, for each gradient line, the gradient transition zone was defined as the distance between the two local minima, when reliably detectable. The reconstructed gradient transition zone is reported in Figure 1e, with the tumour border highlighted through the red circles. Finally, the ZOT was segmented, stemming from the gradient transition zone, as outlined by the red area in Figure 1f. Figure 2 shows two examples of the ZOT outlined as the region between the two green lines, detected for an MVI+ (Figure 2a) and MVI− (Figure 2b) HCC nodule, respectively, with the tumour ROIs superimposed in red.

It is worth noting that the ZOT does not “isotropically” follow the segmented tumour margins, but proceeds along the path characterised by the highest local contrast variations. The ZOT detection was carried out for all patients, referring to the slice with the largest segmented tumour ROI.

All the procedures performed in this study were implemented in MatLab^®^ (R2019b v.9.7, The MathWorks, Natick, MA, USA).

### 2.5. Classification Model Development

**Figure 3:**
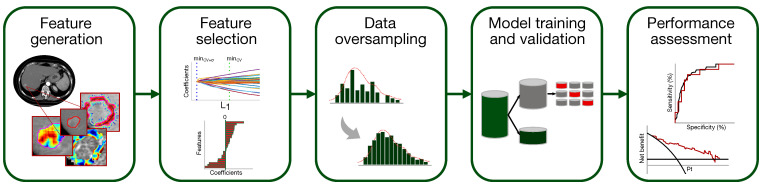
Pipeline of the classification model development for MVI diagnosis.

A classification model to predict the presence of MVI in HCC has been set up according to the flowchart of Figure 3 (the single steps are deepened in Section 2.5.1–Section 2.5.5).

#### 2.5.1. Feature Generation

**Figure 4:**
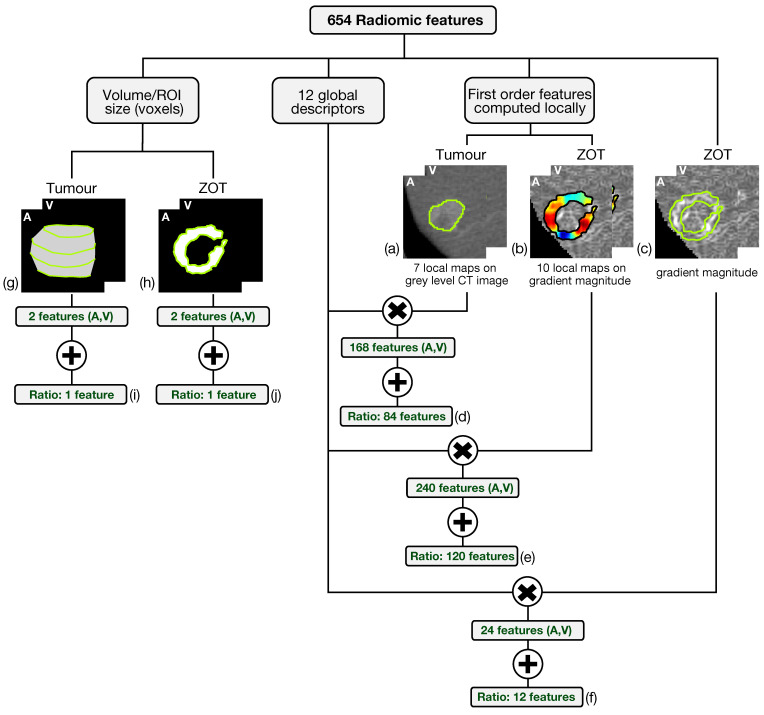
The scheme summarizes the feature generation process. The six-hundred fifty-four radiomic features include 648 features achieved through the computation of 12 global descriptors on different local maps of first order features referred to tumour on grey level CT images (a) and ZOT (b,c) on gradient magnitude images, yielding 84 (d), 120 (e), and 12 (f) features, respectively. In addition, 6 features measure, in pixels, the ROI volume of tumour (g) and ZOT (h) in both arterial (A) and venous (V) phases as well as their ratio (i,j).

Altogether, 654 radiomic features have been generated from both arterial and venous contrast-enhanced CT images by analyzing tumour ROIs and ZOT. The scheme in Figure 4 summarizes the feature generation process.

**Figure 5:**
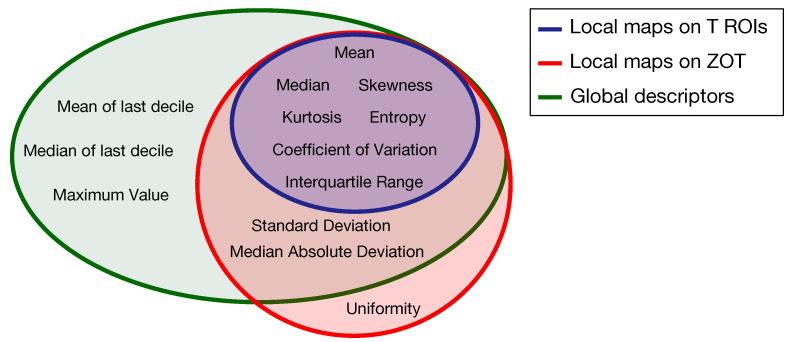
Venn diagram of the radiomics features computed as global descriptors (12, green area) or locally as parametric maps on T ROIs (7, blue area) and ZOT (ten, red area).

Based on the method proposed in a previous paper [30] for computing the local first order radiomics features, 84 features originated from tumour ROIs, computed on the grey level CT images of both the arterial and the venous contrast-enhancement phases. In practice, 7 first order features were computed locally and represented through as many parametric maps, each summarized in single values by 12 global descriptors, therefore giving 84 different features (Figure 4a). In addition, 120 features were generated from the arterial and venous phases, respectively, by computing 10 local first order features on the gradient magnitude images of the ZOT and the same 12 global descriptors as above, on each of them (Figure 4b). Figure 5 depicts the Venn diagram reporting all the radiomics features considered, whether they are computed as global descriptors (green area) or as local parametric maps on T ROIs (blue area) and the ZOT (red area).

Subsequently, 12 global descriptors were also computed directly on gradient magnitude images of the arterial and venous phases within the ZOT (Figure 4c). Moreover, 216 radiomics features stem from the ratio between the features computed in the tumour (i.e., 84 features, Figure 4d) and the ZOT (i.e., 132 features, Figure 4e,f) ROIs, on the arterial and venous phases, respectively. Finally, 4 features refer to the volume measured in voxels of T and the ROI size of the ZOT, separately, in the arterial and the venous phases (i.e., 4 features, Figure 4g,h), together with the ratios of the two couple of features (i.e., referred to arterial and venous phases) for the ZOT and the tumour separately (i.e., 2 features, Figure 4i,j).

#### 2.5.2. Feature Selection

Considering that there were two different ROIs (tumour and ZOT), the Authors chose to finally select no more than four features, roughly two for each region. The most informative radiomic features for diagnosing MVI were selected from the entire set of features generated after data normalisation and standardisation. Data normalisation was carried out by means of contrast stretching using the linear scaling of each single feature histogram between the 2.5 and 97.5% percentiles of the distribution. The features were then standardized. Feature relevance was ranked using the Least Absolute Shrinkage and Selection Operator (LASSO) with the optimal tuning parameter (λ chosen using 3-fold cross-validation (CV), by weighting each observation of the dataset in order to equalise the balance between positive (MVI+) and negative (MVI−) class instances.

The features kept show non-zero LASSO coefficients at the minimum CV error function.

To prevent overfitting in the classification model for MVI diagnosis, an additional subset limited to four features was then searched among all the features coming through LASSO selection.

All possible combinations of four features were initially considered. The combinations in which at least one couple was correlated with the coefficient ρ > 0.3 were then discarded. Finally, the Wilcoxon rank-sum test at a significance level α = 10${}^{-3}$ was carried out and corrected by Holm-Bonferroni for all the remaining combinations to select that one yielding the highest discrimination capability (i.e., the lowest *p*-value).

#### 2.5.3. Dataset Oversampling

The four features arise from processing the initial dataset (ID). In order to increase the statistical significance of the subsets involved in all the development phases of the classifier, the ID was then oversampled. The strategy adopted exploited the univariate kernel density estimation (KDE) of the four radiomic features selected from the ID, combined with the Latin hypercube sampling (LHS) method [31]. KDE was carried out for the MVI+ and MVI− populations separately, starting from the normalised feature distributions. A normal kernel was adopted for KDE, exploiting a non-parametric approach for automatic estimation of the optimal bandwidth, according to the method proposed in [32]. A stratified sampling strategy was adopted for feature oversampling, thus simulating, via LHS, 30 MVI+ and 50 MVI− new instances. Since LHS allows controlling the correlation between oversampled variables, the correlation matrices between the four selected features were computed for the MVI+ and MVI− populations, respectively. Thus, $10^{5}$ runs of LHS were carried out and, for each run the difference matrix between the correlation matrix of oversampled and initial features (matrix $d_{\rho }$) was assessed. The optimal LHS solution has been chosen as the one minimizing the cost function, f(x), defined as the square of the dot product of all entries of $d_{\rho }$, excluding the main diagonal (Equation (1)).

(1) $$f\left(x\right)={\left(\prod_{i,j}d_{\rho_{\left(i,j\right)}}\right)}^{2}\;\;\;j>i,\;\;\;\forall i,j\in \left\{1,2,3,4\right\}$$

Finally, an oversampled dataset (OD) was achieved, made up of 169 samples of which 62 were MVI+ and 107 were MVI− instances. The similarity between the initial and the oversampled distributions of the four features selected was assessed using the mean square error (MSE) between each distribution and the corresponding KDE function.

Hereinafter, all classifier development phases, including training with 3-fold CV and external validation, refer to the OD. However, in order to verify the statistical representativeness of the OD with respect to ID, two preliminary MVI diagnostic models were developed on the ID and the OD respectively, without carrying out external validation, and were compared in order to check their equivalence. In this regard, details are provided in Supplementary Materials.

#### 2.5.4. Support Vector Machine (SVM) Training and Validation

An SVM classifier with a linear kernel was adopted to develop a diagnostic model for MVI in which MVI+ instances were considered to be the True Positive (TP) and the MVI− instances were True Negative (TN). The OD was split into a training set made up of 117 samples, 43 MVI+ and 74 MVI−, and a test set for external validation of 52 samples, 19 MVI+ and 33 MVI−, thus maintaining the original proportion in each subset of positive and negative instances.

The SVM training was carried out using 100 runs of 3-fold CV for internal validation. For each run, a random seed was used to split the training set into 3 folds, each of 14 MVI+ and 25 MVI− instances. To allow for class imbalance, the misclassification cost (C) of the SVM classifier was scaled using the prior probability of each class [33].

After each SVM training session, the predicted class of each sample and its corresponding probability score, namely the radiomic score, were estimated using a binomial logit function. In addition, the receiver operating characteristics (ROC) curve was built for each trained SVM classifier, and the corresponding area under the curve (AUC) was computed. Thus, the models most prone to overfitting, yielding an AUC on the internal validation set higher than that on the corresponding training set were discarded and, for each run of 3-fold CV, just the SVM model with the highest AUC on the internal validation set was kept.

Hence, the selected models, at most 100 competitors, were trained on the entire training set. The final SVM classifier was chosen to be the one with the highest AUC and informedness (I) regarding the training set. Finally, the classifier was externally validated on the test set.

#### 2.5.5. Performance Assessment

The performance of the final radiomic model was assessed using AUC, sensitivity (SN), specificity (SP), and I measured at the Youden cut-off. In addition, the radiomic scores achieved on both the training and the test sets were represented using waterfall plots. The median and interquartile ranges (IQRs) of the radiomic score were computed in the MVI+ and the MVI− classes for both the training and the test sets. Median differences were statistically assessed using the Wilcoxon rank-sum test (α = 10${}^{-3}$).

To assess model performance on new clinical populations in which errors are unknown, the negative predictive value (NPV), positive predictive value (PPV), negative predictive ratio (NPR), positive predictive ratio (PPR), and the predictive summary index (PSI), defined as PSI = PPV + NPV − 1 [34], were considered.

Moreover, the calibration curve of the classifier was drawn referring to the test set and was used in combination with the Hosmer-–Lemeshow test (α=0.05) to assess the goodness-of-fit of the radiomic model, that is, the agreement between the predicted and the measured MVI risk probabilities. Finally, to assess the clinical usefulness of the radiomic model, decision curve analysis (DCA) [35] was used, thus computing the net benefit at different threshold probabilities in the test set.

## 3. Results

### 3.1. Baseline Patient Characteristics

The baseline characteristics of the study population are detailed in Table 1. There were 89 tumours, 32 MVI+ and 57 MVI−, preoperatively detected by CT in 78 patients.

### 3.2. Radiomics Feature Selection and Data Oversampling

**Figure 6:**
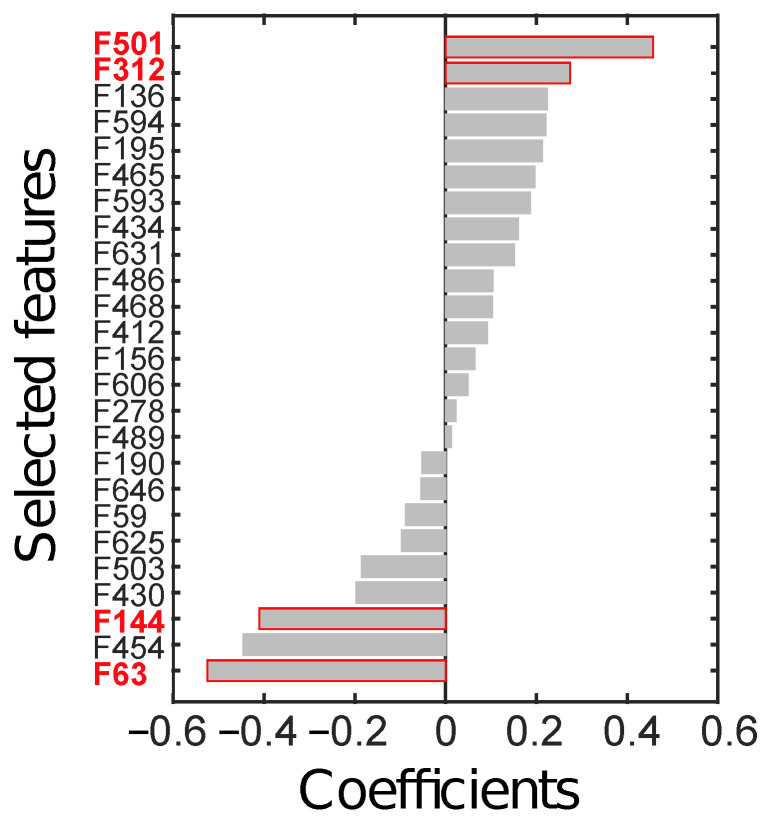
Radiomics features (F) selected through LASSO, sorted by their coefficients. The four features in red text colour (i.e., F63, F144, F312, and F501) are the ones finally selected to constitute the radiomics signature.

Feature selection, performed on the ID through LASSO, yielded 25 relevant radiomics features, reported in Figure 6 according to their rank.

From all possible combinations of four features (i.e., 12650 combinations), a total of 2788 were discarded due to their high correlation. Finally, 4 combinations were significant at the Wilcoxon rank-sum test (*p* < $10^{-7}$), after considering the Holm-Bonferroni correction. The most discriminant combination (*p*∼10${}^{-8}$) was composed of those features with the highest absolute weight, the identifier of which is highlighted in red in Figure 6. In particular, F63 is the skewness computed on the parametric maps of local skewness (S-s) in the ZOT during the arterial phase (hereinafter, S-s [ZOT,A]). F144 refers to the entropy computed on the local entropy map (E-e), calculated in the tumour ROI during the arterial phase (hereinafter, E-e [T,A]). F312 is the entropy of the local uniformity (U-e) computed in the ZOT during the venous enhancement CT phase (hereinafter, U-e [ZOT, V]). Finally, F501 is the ratio between S-s in the ZOT computed during the arterial and during the venous phases (hereinafter, S-s [ZOT, A/V]).

**Figure 7:**
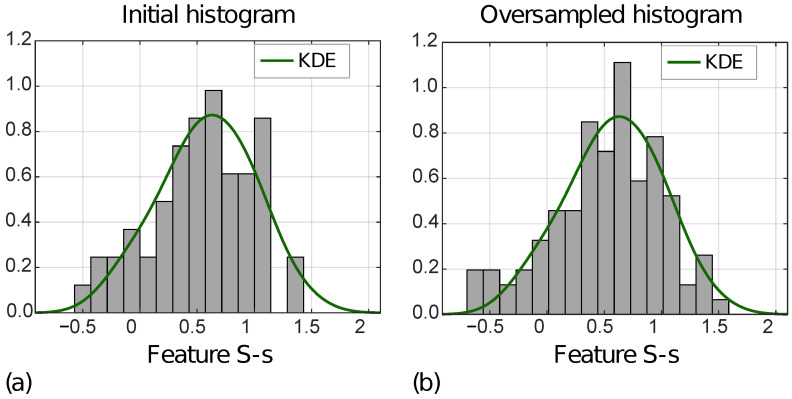
Normalized histograms of feature S-s [ZOT,A] (i.e., F63) referred to ID (**a**) and OD (**b**) for MVI− class.

Figure 7 shows the distribution of the S-s [ZOT,A] for MVI− instances in the ID (a) and OD (b), with the KDE function superimposed.

In addition, Table 2 reports MSEs computed between the KDE functions, and the initial and the oversampled feature distributions, respectively, for MVI+ and MVI− instances, separately. For all four features, the MSEs decreased from the ID to the OD.

### 3.3. Radiomics Signature and Classifier Performance

Of the 100 competing models stemming from the internal validation, 16 showed equivalent performance, with the highest I regarding the training set, being 0.69. The radiomics signature, g(x), for diagnosing MVI, as defined by the ultimately selected SVM classifier, is reported in Equation (2):

(2) g(x)=−0.004−0.519·S-s[ZOT,A]−0.714·E-e[T,A]+0.718·U-e[ZOT,V]+0.872·S-s[ZOT,A/V]

**Figure 8:**
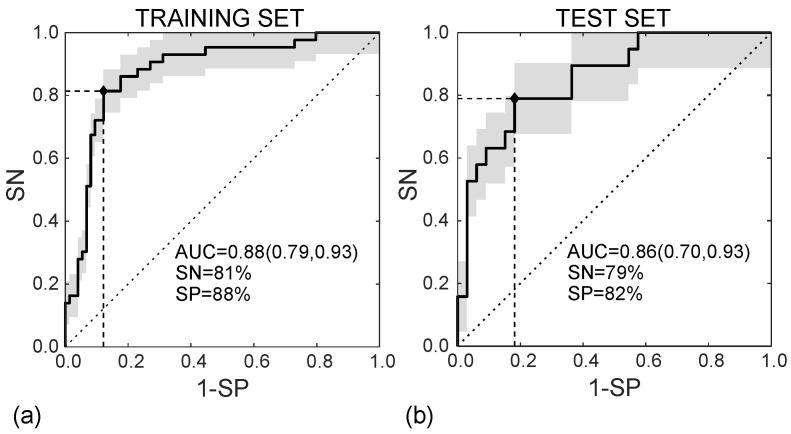
ROC curves referred to the training (**a**) and test (**b**) sets, achieving AUC = 0.88 95%CI (0.79, 0.93) and AUC = 0.86 95%CI (0.70, 0.93), respectively.

In the training set, the SVM classifier diagnoses the MVI presence in HCC nodules according to the ROC curve shown in Figure 8a, with AUC = 0.88 (95%CI, (0.79–0.93)), SN = 81%, and SP = 88% (I = 0.69). In the test set, diagnosing MVI was carried out according to the ROC curve in Figure 8b, with AUC = 0.86 (95%CI (0.70–0.93)), SN = 79%, and SP = 82% (I = 0.61).

**Figure 9:**
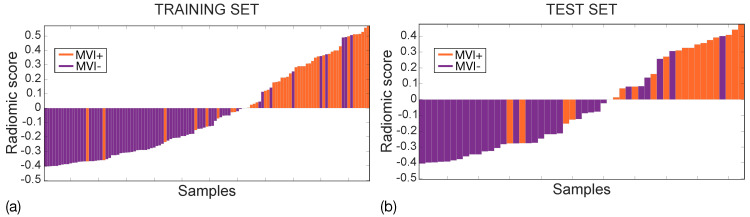
Waterfall plots for training (**a**) and test (**b**) sets. MVI+ and MVI− samples are represented through orange and purple bars, respectively with zero representing the cut-off.

Figure 9 shows the waterfall plots of the radiomic scores achieved on the training (a) and the test (b) sets, with orange and purple bars representing MVI+ and MVI− instances, respectively, with zero representing the cut-off. The radiomic score differs between MVI+ and MVI− instances with *p*∼10${}^{-11}$ and *p*∼10${}^{-5}$ in the training and test sets, respectively, with higher values in MVI+ class, indicating more intratumour and ZOT heterogeneity. Indeed, the median MVI+ radiomics scores were 0.284 (IQR = 0.355) and 0.310 (IQR = 0.344) while the median MVI− radiomics scores are −0.276 (IQR = 0.240) and −0.273 (IQR = 0.286), in the training and test sets, respectively. The MVI diagnosis was achieved with 9 False Positive (FP) and 8 False Negative (FN) in the training set (117 samples), and 6 FP and 4 FN in the test set (52 samples). Accordingly, the SVM classifier in the training set showed an NPV = 89% and PPV = 80%, with NPR = 0.2 and PPR = 7.3 while, in the test set, NPV = 87% and PPV = 71%, which led to an NPR = 0.3 and a PPR = 5.5. Finally, the PSI was equal to 0.69 and 0.58 in the training and the test sets, respectively.

The SVM performance regarding the training and the test sets is reported in Table 3.

### 3.4. Clinical Benefit of the Radiomics Model

**Figure 10:**
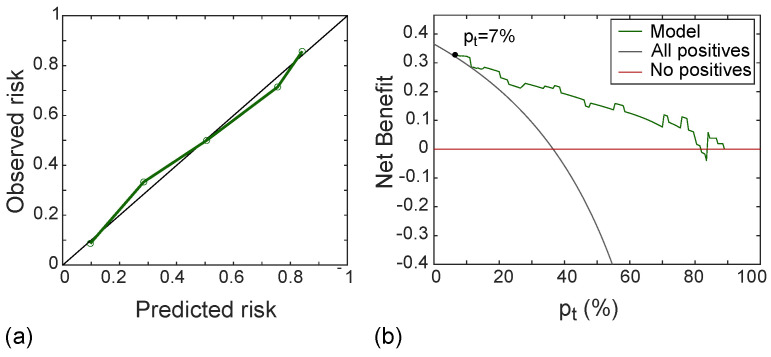
Calibration line (**a**) yielding *p*-value at Hosmer–Lemeshow test equals to 0.558 and DCA (**b**) with a net benefit of 32% at p${}_{t}$ = 7%, referred to the test set.

Figure 10a shows the calibration curve of the radiomic signature in the test set, with good agreement between predicted and observed risk probabilities. The Hosmer–Lemeshow test yielded *p* = 0.556, which indicates no departure from the perfect fit. In addition, the DCA shown in Figure 10b indicated that the radiomic model was better than both “all positive” and “all negative” strategies if the threshold probability was over 7%, which corresponded to a maximum net benefit of 32%.

## 4. Discussion

The present study identified a CT-based radiomic model developed and validated for the prediction of the MVI of HCCs ≤ 3.0 cm in diameter. The present classifier is a very easy-to-use tool, made up of only four features, mainly referring to ZOT properties. The radiomic signature achieved a very good diagnostic performance in the early detection of MVI.

Some recent studies ([20,21,22,23,24,36]) have proposed radiomic models for MVI prediction, exploiting contrast-enhanced CT imaging. Unfortunately, these studies presented several limitations. The first was the absence of an ad-hoc analysis concerning nodule dimension in these series. This greatly limits the clinical usability of these results as, especially in the era of expanding imaging surveillance, it is increasingly likely that liver lesions will be identified at a very early diagnostic stage in the future. And, to date, it is doubtful that the results of these studies could have the same diagnostic performance even for small lesions. Another limitation was that all these studies exploited a very high number of radiomic features when compared to the sample size of their validation datasets. From a methodological point of view, this makes the risk of overfitting very high, thus jeopardising the statistical and clinical significance of their results. Finally, almost all the studies ([21,22,23,24,36]) showed lower predictive performance rates as compared with those in the present study. The only study [20] showing a better performance better than the present study did not report the number of finally selected radiomic features.

Unlike the above-mentioned series, the present study analysed only small lesions (nodules ≤ 3.0 cm) for two reasons. First, as reported above, the lesions detected during surveillance program in patients at high risk of developing HCC are small in size. Second, the majority of the therapeutic choices available for patients in the very early and early stages of the BCLC staging system [14] were based on the patient’s clinical conditions and on the tumour burden (tumour size and number). In particular, except for single lesions, the therapeutic decisions in the very early and early stages concern nodules ≤3.0 cm. Furthermore, the present study considered only four radiomic features as the minimum subset size to classify MVI+ and MVI− HCC nodules, thus minimising the risk of overfitting while preserving the goodness of prediction of MVI status. Nonetheless, it was also decided to increase the representativeness of the training and test sets by carrying out data oversampling. This was accomplished by generating new feature values (rather than new image data). Finally, the authors’ choices allowed achieving the highest rates of diagnostic performance in predicting MVI in HCC as compared with published series [20,21,22,23,24,36]).

As a matter of fact, the highest percentage of MVI in HCC occurs in the peritumoural regions, approximately within 1 cm from the tumour margins [12]. For this reason, the majority of the previous studies included the peritumour in the radiomic analysis [21,23,24]. The peritumour area is generally a priori defined at 3 mm ([24]), 5 mm ([21]), or 1 cm ([23]) distance from the tumour margins, by applying dilation on segmented tumour ROIs. The fixed distance from the tumour border produces an isotropic area which scarcely reflects the region in which the microvascular infiltration of the tumour occurs. In addition, this area is intrinsically dependent on the manual definition of the tumour profile by the radiologist, thus being dependent on the limited accuracy of the human eye. An additional difficulty arises from the uncertainty of the HCC profile on CT, especially for small lesions. Therefore, the absence of a clear imaging distinction between the tumour and the peritumoural parenchyma also requires focusing on the ZOT in which the most information regarding functional and structural tissue modifications should be retained. To carry out a dedicated radiomic analysis on the ZOT, the information in the proximities of the HCC nodule was exploited, using an adaptive nodule-driven approach. Hence, the ZOT is detected by searching for the areas alongside the tumour borders where CT images have the highest density of local contrast variations. Of course, the resulting analysis is anisotropic, thus giving the ZOT an irregular shape. The new approach for analysing the peritumoural inflammatory region makes the information derived from the ZOT crucial for distinguishing MVI+ from MVI− HCC nodules. In fact, three out of four of the finally selected features in the radiomic signature retain information from the ZOT whether they refer to arterial or venous contrast-enhanced CT phases. It should be noted that these vascular imaging phases are those recommended by the guidelines [14] for reaching the imaging diagnosis of HCC.

All the features in the radiomic signature (Equation (2)) depict different aspects of tumour and ZOT heterogeneity. As highlighted by the waterfall plots in Figure 9, high radiomics scores characterize MVI+ nodules, whilst low scores characterize MVI−. In general, to explain which are the main properties differentiating HCC nodules with and without MVI, it is necessary to under- stand the meaning of each single features, separately. Starting from F144, that is E-e [T,A], it represents tumour heterogeneity measured during the arterial enhancement CT phase. This feature assumes higher values in MVI+ than MVI− group. This result is likely due to the high number of unpaired arteries developed into the lesion during the de-differentiating process of the hepatocarcinogenesis. As regards F312, that is U-e [ZOT, V], it still is a measure of tissue heterogeneity, referring to ZOT during the venous enhancement CT phase. Probably this parameter reflects the heterogeneity of the contrast distribution in the extravascular space due to the presence of microvascular infiltration that produce a lower interstitial hyperdensity near the thrombotic vessels. Indeed, F312 has lower values in MVI− than in MVI+ group, meaning that HCC nodules without MVI have homogeneous image contrast within ZOT, in the proximity of tumour borders, where no relevant differences are detected between inner and outer tumour regions. Moreover, F63, that is S-s [ZOT, A] characterizes the distribution of local areas with high and low image contrast during the arterial phase within ZOT. According to the values assumed, F63 shows that in ZOT of MVI+ nodules there is a predominance of areas characterized by strong variations of image contrast. Of course, this represents one more heterogeneity measure, although of a different type. So far, we have seen that MVI+ HCC nodules have a higher intra-tumour heterogeneity, besides being characterized by the strongest differences between tumour and peritumour yielding a high ZOT heterogeneity. Finally, F501, S-s [ZOT, A/V], is the ratio of F63 computed during the arterial and venous phases, respectively. Accordingly, it is somehow correlated to the time evolution of F63. Basically, the higher values of F501 in MVI+ than MVI− group mean that, when comparing arterial and venous CT enhancement phases, MVI+ HCC nodules report large variations between the two phases, whilst MVI− HCC nodules seems keeping most similar to each other. In the presence of a MVI+ status, this feature discloses the temporal heterogeneity of HCC nodules between the arterial and vascular CT phases, which may hint at altered wash-out vascular properties of MVI+ nodules probably due to the vascular infiltration.

Our radiomics model demonstrated a good reliability for diagnosing MVI in small HCC, in terms of PPV, NPV, PPR, NPR, and PSI values. In particular, NPV, the most relevant parameter to select the best candidates for surgical treatments and liver trans- plantation, is very high (87%). In addition, the low NPR (i.e., NPR < 1) and high PPR (i.e., PPR ≫ 1) values in both training and test sets, indicate a high effectiveness of the classifier in performing a correct diagnosis of either MVI− or MVI+ nodules, which is far most frequent than the diagnosis failure.

It is well known that the heterogeneity of the antigenic constellation in HCC suggests an antigenic mosaicism, which can be expressed as synchronous or metachronous, depending on the tumour degree of differentiation [37]. Therefore, it is important to evaluate the entire nodular volume to enhance the possibility of MVI detection in any segmental portions of the lesion that expresses mosaicism. Moreover, in the future it will be important to adopt an improved surveillance program, for example based on the use of MRI, in order to identify small lesions [38,39] and analyse MVI in these small nodules, hoping that the grade of differentiation is the same for all patients who underwent surveillance for high risk of developing HCC.

A recent experience suggests an association of LIRADS-5 (LR-5) class with unfavourable pathological characteristics of resected HCC, such as MVI, satellitosis and capsule infiltration, with a significant negative impact on postoperative oncological outcomes [40]. Unfortunately, this approach could not be applied to our study due to two major limitations. The first one is related to the intrinsic limitations of LIRADS system, not jet accepted in the EASL guidelines for the management of HCC [14,41]. The second important limitation arises from the nature of the mentioned study focused on the statistical associations between LR-5 and prognostic negative features such as MVI. It will be important to design a study to understand how the imaging features which determine the classification LR-5 could correlate with MVI in order to identify the real pathophysiological associations between imaging features and negative prognostic factors.

Our results, if confirmed in a prospective study, could improve the daily clinical practice for the management of patients with HCC. In fact, the use of radiomics could help radiologists in the assessing the presence of MVI also for small lesions. In the era of tailored medicine, this could help hepatologists in the decision making for patients in very early and early stage, choosing the most effective treatments for each patient. Furthermore, many other studies could be planned to understand which the best treatment strategy will be to approach a small lesion with MVI positivity. For example, knowing the MVI positivity of a HCC could encourage future studies combining locoregional treatments such as chemoembolization with systemic therapies such as immune checkpoint inhibitor plus tyrosine kinase inhibitor. Moreover, it is well known that, in hepatology, a major issue is the choice of chemoembolization repetition and the optimum number of chemoembolization sessions before switching to another treatment or best supportive care [42]. Probably, the effectiveness of this treatment in some cases is low due to MVI positivity and this is the reason why many proposed scores such as the ART score were not found to work as objective tools to guide chemoembolization retreatment in all series and populations [42]. For all these reasons, the combination of chemoembolization with systemic therapies such as immune checkpoint inhibitor plus tyrosine kinase inhibitor in MVI positive patients with HCC could favour overcoming these limitations.

There are three main limitations in this study. The first one is the small sample size of the ID, arising from strict inclusion criteria, which required CT imaging of HCC nodules ≤3 cm in diameter. A future multicentre study will increase the study population, especially to have more MVI+ instances. The second limitation refers to the retrospective nature of the study. Then, another limitation is the lack of histopathological data to be compared with those functional and structural properties of HCC nodules, which have been highlighted from the analysis of the radiomics features meaning in tumour and ZOT, for MVI+ and MVI− groups. A future prospective study will address this issue.

## 5. Conclusions

The present study developed a radiomics classification to early predict the MVI status in HCC nodules of at most 3.0 cm in diameter, achieving the highest diagnostic performance rates available in the literature. For the first time, this work presents a ZOT detection of HCC nodules, whose analysis resulted to be crucial in MVI diagnosis. Our findings show that a strong spatial and temporal ZOT radiomics heterogeneity captures the underlying structural modifications occurring between tumour and normal tissues, this representing a clear sign predictive of a MVI+ status. Finally, the radiomics classifier we identified, using only four features, could represent an easy-to-use tool in the clinical practice for the MVI diagnosis in small HCC. 

## Figures and Tables

**Table 1:** Baseline Characteristics of the study population.

Variable	Value
**Age (y) ${}^{1}$**	63 (53–73)
**Mean (y) ${}^{2}$**	62.91(±11.17)
**Male sex**	59 (75.6)
Age (y) ${}^{1}$	62 (50–73)
Mean age (y) ${}^{2}$	61.51 (±11.31)
**Female sex**	19 (24.3)
Age (y) ${}^{1}$	66 (64–73)
Mean age (y) ${}^{2}$	67.67 (±9.48)
**Hepatitis C infection**	55 (73.3)
**Hepatitis B infection**	12 (16)
**Other disease origin**	11 (14.7)
**AFP**	236.1 (4–57)
**Total bilirubin level (mg/dL)**	0.89 (0.60–0.96)
**Serum albumin level (g/dL)**	4.11 (3.9–4.5)
**International normalized ratio**	1.15 (1.5–1.2)
**Platelet count (×10${}^{3}$/μL)**	142.9 (91–175.75)
**Model for End-Stage Liver Disease score**	7 (6–8)
**Child-Pugh class**	
5	52 (66.7)
6	21 (26.9)
7	2 (2.6)
8	3 (3.8)
**Presence of cirrhosis**	55 (70.5)
**Mean diameter of largest tumour (mm)**	21.06 (17–26)
**No. of tumors/patient**	
Solitary	68 (87.2)
Two tumors	9 (11.5)
Three tumors	1 (1.3)
**Extension of hepatectomy**	
Single or multiple wedges	49 (62.8)
Segmentectomy	11 (14.1)
Bisegmentectomy	7 (9.0)
Major hepatectomy	11 (14.1)

Unless otherwise indicated, data are number of patients, with percentage in parentheses. ^1^ Data are continuous variables, reported as medians with IQRs in parentheses. ^2^ Data are mean ± standard deviations.

**Table 2:** Mean square errors (MSEs) between kernel density estimation (KDE) functions and initial and oversampled histograms of the four selected radiomics features, which refer to both tumour (T) regions of interest (ROIs) and zone of transition (ZOT) of arterial (A) and venous (V) phases.

Feature	MVI+		MVI−	
	Initial	Oversampled	Initial	Oversampled
F63: S-s [ZOT,A]	0.038	0.014	0.039	0.017
F144: E-e [T,A]	0.227	0.115	0.259	0.074
F312: U-e [ZOT,V]	1.579	0.494	0.264	0.092
F501: S-s [ZOT, A/V]	10${}^{-4}$	10${}^{-5}$	10${}^{-6}$	10${}^{-6}$

**Table 3:** Support vector machine (SVM) classifier performance for microvascular invasion (MVI) diagnosis in training and test sets.

	Training Set	Test Set
SN	81%	79%
SP	88%	82%
I	0.69	0.61
NPV	89%	87%
PPV	80%	71%
NPR	0.2	0.3
PPR	7.3	5.5
PSI	0.69	0.58

## Data Availability

The data are not available because of patient privacy.

## Supplementary Materials

The following are available online at https://www.mdpi.com/article/10.3390/cancers14071816/s1, Figure S1: Median ROC curves achieved for training (a) and test (b) referring to ID. The vertical lines indicate the MAD of TPR values, the red triangle highlights the Youden cut-off. Figure S2: Median ROC curves achieved for training (a) and test (b) referring to OD. The vertical lines indicate the MAD of TPR values, the red triangle highlights the Youden cut-off. Figure S3: Comparison of between ROC curves of ID and OD, referring to training (a) and test (b) sets, respectively.

## Abbreviations

The following abbreviations are used in this manuscript:

AUC	Aurea under the curve
BCLC	Barcelona clinic liver cancer
CT	Computed tomography
CV	Cross-validation
DCA	Decision curve analysis
EASL	European Association of the Study of the Liver
HCC	Hepatocellular carcinoma
ID	Initial dataset
IQR	Interquartile range
KDE	Kernel density estimation
LT	Liver transplantation
LASSO	Least absolute shrinkage and selection operator
LHS	Latin hypercube sampling
MSE	Mean square error
MVI	Microvascular invasion
NIH	National Institutes of Health
NPR	Negative predictive ratio
NPV	Negative predictive value
OD	Oversampled dataset
PPR	Positive predictive ratio
PPV	Positive predictive value
PSI	Predictive summary index
ROC	Receiver operating characteristics
ROI	Region of interest
SN	Sensitivity
SP	Specificity
SVM	Support vector machine
TP	True positive
TN	True negative
ZOT	Zone of transition
