# Supplementary material for: Automatically Extracted Machine Learning Features from Preoperative CT to Early Predict Microvascular Invasion in HCC: The Role of the Zone of Transition (ZOT)
Source: Cancers (Basel). 2022 Apr 3;14(7):1816. doi: 10.3390/cancers14071816 (PMC8997857; doi:10.3390/cancers14071816)
Supplement: Supplementary file 1 [file cancers-14-01816-s001.zip › cancers-1628127-SI.pdf]

Automatically extracted machine learning features from preoperative CT to early predict microvascular invasion in HCC: the role of the Zone of Transition (ZOT)

SUPPLEMENTARY MATERIALS

1. Preliminary ML models

To verify the statistical representativeness of the Oversampled Dataset (OD) with respect to Initial Dataset (ID), two preliminary MVI diagnostic models were developed on the ID and the OD respectively, without carrying out external validation, and were compared for checking their equivalence. In particular, ID and OD were randomly split into training and test sets, made by 60 and 29 samples (as for ID) and 113 and 56 (as for OD), respectively. Then, 100 runs of 3-fold Cross-Validation (CV) were performed using the Support Vector Machine (SVM) classifier to predict MVI+ instances. After each SVM training session, the predicted class of each sample and its corresponding probability score, namely the radiomic score, were estimated using a binomial logit function. In addition, the Receiver Operating Characteristics (ROC) curve was built for each trained SVM classifier, and the corresponding Area Under the Curve (AUC) was computed. The models most prone to overfitting, yielding an AUC on the test set higher than that on the corresponding training set, were discarded and, for each run of 3-fold CV, only the SVM model with the highest AUC on the internal validation set was kept. At the end, there were 100 competing models and their median ROC curve was built, by considering the Median Absolute Deviation (MAD) to assess the True Positive Rate (TPR) dispersion for each False Positive Rate (FPR) value. The same procedure was replicated for the 100 ROC curves of the test sets, thus finally achieving four median ROC curves referred to training and test sets of ID and OD, respectively, to be checked for equivalence.

2. Checking the equivalence of initial and oversampled datasets

First, performance on training and test sets of both ID and OD were assessed considering the AUC and the couple of sensitivity (SN) and specificity (SP) measured at the Youden cut-off (YI). Then, in order to check for equivalence, the median ROC curves of ID-referred training and test sets, were linearly interpolated and evaluated at all FPR values of the OD-referred ROC curves in training and test sets. Then, a two-tail t-test was performed to state their equivalence, using $\alpha=10^{-3}$ significance level.

3. Results

Fig. 1 shows the ROC curves achieved on training (a) and test (b) sets with respect to the ID, where vertical lines indicate MAD values of each (TPR, FPR) couple. In addition, the red triangle highlights the Youden cut-off. In training and test sets, ID achieves AUC=0.87 (95% CI, 0.77-0.98) and AUC=0.82 (95% CI, 0.65-0.99), respectively, with SN=86% and SP=79% in training (YI=0.65) and SN=82% and SP=79% in test (YI=0.61). Similarly, Fig. 2 reports the ROC curves achieved on training (a) and test (b) sets referring to OD. Then, AUC=0.88 (95% CI, 0.81-0.96) with SN=85% and SP=81% in training (YI=0.66) and AUC=0.84 (95% CI, 0.73-0.96) with SN=80% and SP=78% in test (YI=0.58), respectively. Finally, Fig.3 shows the comparison between ROC curves referred to OD (light blue lines) and the interpolated referred to ID (purple line) in training (a) and test (b) sets, which resulted statistically equivalent with p-value=0.39 and p-value=0.12 for training and test sets, respectively. This ultimately confirms the representativeness of the OD with respect to the ID.

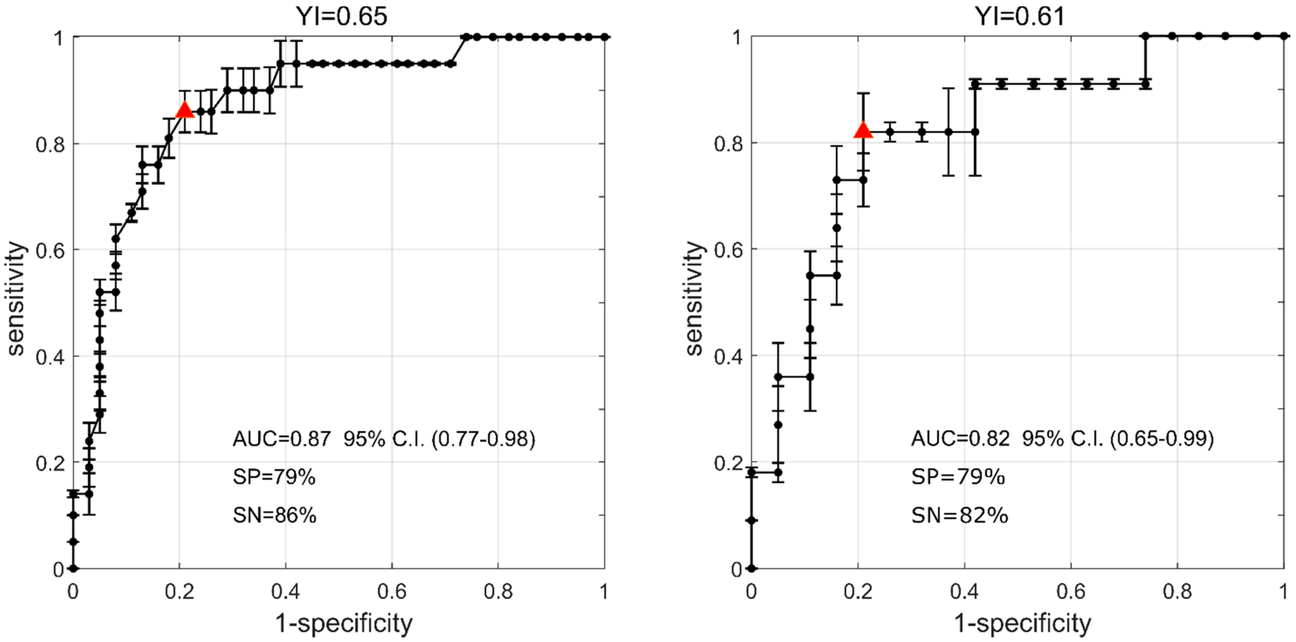

(a) (b)

Figure S1. Median ROC curves achieved for training (a) and test (b) referring to ID. The vertical lines indicate the MAD of TPR values, the red triangle highlights the Youden cut-off.

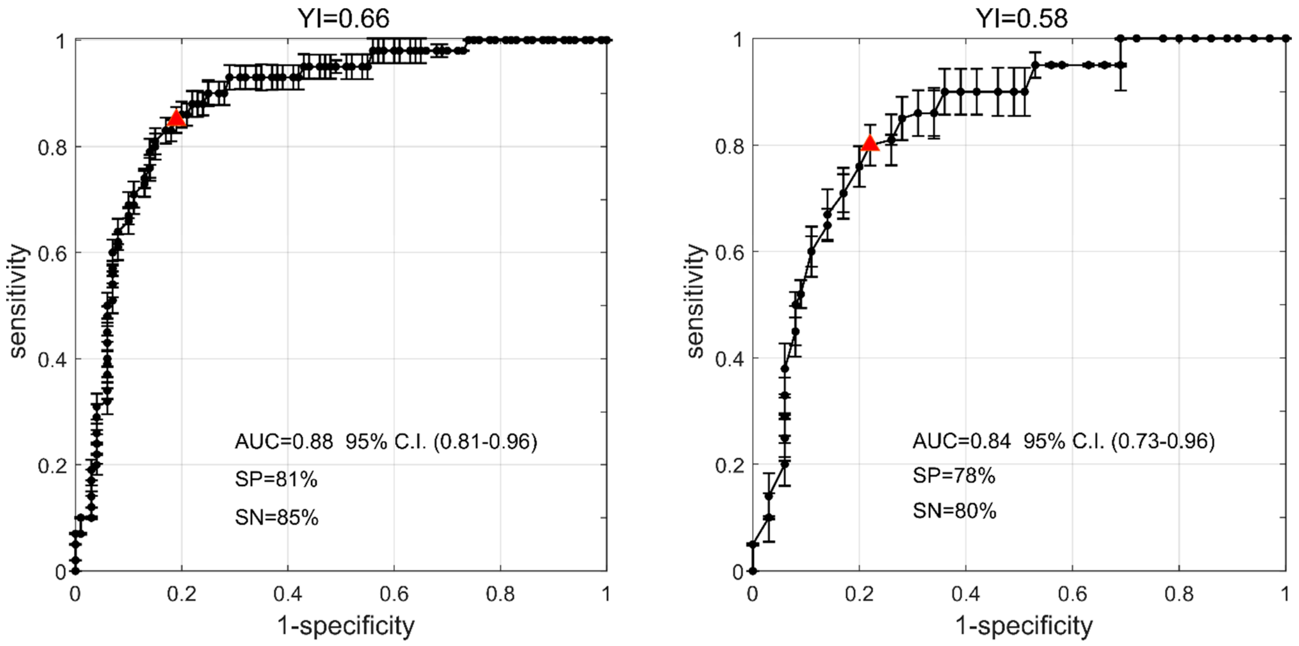

(a) (b)

Figure S2. Median ROC curves achieved for training (a) and test (b) referring to OD. The vertical lines indicate the MAD of TPR values, the red triangle highlights the Youden cut-off.

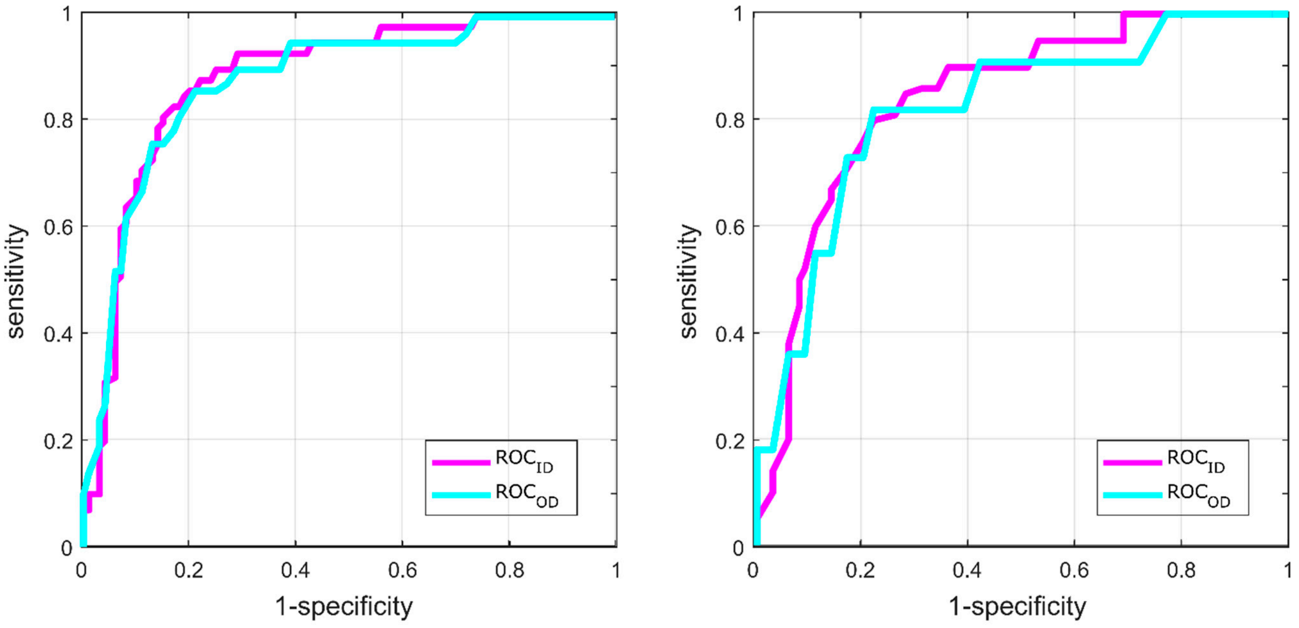

(a) (b)

Figure S3. Comparison of between ROC curves of ID and OD, referring to training (a) and test (b) sets, respectively.
